# A comparative study of the population genetics of wild and cultivated populations of *Paris polyphylla* var. *yunnanensis* based on amplified fragment length polymorphism markers

**DOI:** 10.1002/ece3.5589

**Published:** 2019-08-20

**Authors:** Yu Huang, Nong Zhou, Min Yang, Yuxiang Shen, Dequan Zhang

**Affiliations:** ^1^ College of Food and Biology Engineering The Chongqing Engineering Laboratory for Green Cultivation and Deep Processing of the Three Gorges Reservoir Area's Medicinal Herbs Chongqing Three Gorges University Chongqing China; ^2^ College of Pharmacy and Chemistry Dali University Dali China; ^3^ The Agricultural College Anshun University Anshun China

**Keywords:** amplified fragment length polymorphism markers, cultivation, genetic diversity, medicinal plants, *Paris polyphylla* var. *yunnanensis*, UPGMA dendrogram

## Abstract

*Paris polyphylla* var. *yunnanensis* is one of the original plants used to make the traditional medicine Paridis Rhizoma. Wild individuals have been excessively collected in recent decades, and thus, it has become increasingly endangered. Cultivation is a major method for the conservation and sustainable utilization of its wild resources. In this study, amplified fragment length polymorphism markers were used in the genetic analysis of 15 wild and 17 cultivated populations of *P. polyphylla* var. *yunnanensis*. This study revealed that cultivated populations possessed higher genetic diversity than wild ones at the species level (*H* = 0.2636 vs. 0.2616, respectively). However, most of the genetic variation was found within populations for both of these groups (Φ_ST_ = 18.83% vs. 19.39%). In the dendrogram produced using UPGMA, the 32 populations were divided into three groups (I, II, and III). In group II, both wild and cultivated populations were included, but remained in distinct clusters within this group, which showed the significant separation between the cultivated and wild populations. Furthermore, wild populations were also clustered into three subgroups (W‐I, W‐II, and W‐III), with an obvious geographic structure. In contrast, although cultivated populations were similarly placed in three subgroups (C‐I, C‐II, and C‐III), the latter two of these were not separated based on geography. Finally, the wild populations in Guizhou, China (W‐I), possessed higher genetic diversity than those in Yunnan (W‐II and W‐III). As *P. polyphylla* var. *yunnanensis* is an endangered medicinal plant, the fact that it showed richer genetic diversity in its wild populations in Guizhou means that these should be regarded as priority areas for protection and used for provenance selection. Moreover, cultivated populations also showed high genetic variation, which might be attributed to them having originated from mixed provenances, indicating that screening for superior provenances should be carried out as soon as possible.

## Graphical abstract

*Paris polyphylla* var. *yunnanensis* is an important medicinal and endangered plant. This study explored genetic diversity and structure of its wild and cultivated populations.

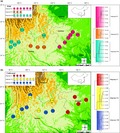

## 1 INTRODUCTION

More than 80% of world's population relies on plant‐based medicines for their primary healthcare needs, most of which come from wild resources (Vines, 2004). Due to increasing demand for such medicines, wild individuals have been overexploited for the last few decades, and many medicinal species have been listed as endangered species, such as *Phellodendron amurense* (Yang et al., 2016) and *Taxus wallichiana* (Gao et al., 2007). To protect wild resources, many countries encourage the cultivation of wild medicinal plants so that they can be used sustainably (WHO, IUCN, & WWF, 1993). Cultivation is an effective means of conservation that is not only regarded as an important reservoir of genetic diversity (Miller & Schaal, 2005), but also as a means to meet the growing demand for traditional medicines (Guo, Lu, Wu, Chen, & Zhou, 2007). Previous studies showed that a long history of domestication in cultivated plants has generally resulted in reductions in their genetic variation, as well as bottleneck effects, such as those that are inferred to have affected soybean (Hyten et al., 2006) and wheat (Haudry et al., 2007). Until now, most studies have focused on economically valuable crops with a long‐term history of cultivation (Doebley, Gaut, & Smith, 2006), but only a few studies have been performed on medicinal species domesticated over the past few decades (Shi, Yang, Chen, & Guo, 2008; Yuan et al., 2010). Comparative studies of the population genetics of wild and cultivated populations would be beneficial to the development of strategies for conserving wild resources, as well as to help in screening populations for superior provenances for use in cultivation.

The genetic impacts of cultivation could be revealed by detecting changes in genetic diversity and structure between wild and cultivated populations using molecular markers, such as restriction fragment length polymorphism (RFLP), random amplification of polymorphic DNA (RAPD), inter‐simple sequence repeats (ISSR), amplified fragment length polymorphism (AFLP), simple sequences repeats (SSR), and single nucleotide polymorphism (SNP) markers, among others. Among these, RFLP (Botstein, White, Skolnick, & Davis, 1980), RAPD (Williams, Kubelik, Livak, Rafalski, & Tingey, 1990), and ISSR (Zietkiewicz, Rafalski, & Labuda, 1994) markers have advantages due to their low costs and simplicity of use, but poor reproducibility restricts their application (Gorji, Poczai, Polgar, & Taller, 2011; Karp, Seberg, & Buiatti, 1996; Navajas & Fenton, 2000). Over the last decade, AFLP and SSR markers, which possess high degrees of polymorphism and good reproducibility, have been widely used in population genetics studies (Mba & Tohme, 2005; Vos et al., 1995). There are obvious advantages and disadvantages to both of these marker types. Compared with SSR marker, AFLP markers, which are dominant, cannot distinguish heterozygotes from homozygotes (Mba & Tohme, 2005), but they can enhance the resolution achieved in population assignment, especially among weakly differentiated populations (Campbell, Duchesne, & Bernatchez, 2003). Further, primers for AFLP marker are universal among species, whereas primers for SSR marker need to be developed for each specific species or genus and are generally not suitable for use in other taxa. In preliminary experiments done before this study, both AFLP and SSR markers were adopted to detect genetic diversity; however, the results revealed that AFLP markers were obviously better than SSR ones in terms of the success rates they achieved and the polymorphism detected with them (Song, Li, Xu, Zhao, & Chen, 2015). SNP markers are third‐generation molecular markers that have high genetic stability and diversity, but their use requires advanced technology and has high costs (Xu, Wang, Hou, & Li, 2015). Thus, AFLP markers are still practical markers to use when evaluating the genetic diversity and population structure of endangered or medicinal plants.

*Paris* L. is an important genus in the family Melanthiaceae of the order Liliales, which includes 27 species and more than 10 varieties (Li, Su, Zhang, & Yang, 2015). Among these species, *Paris polyphylla* Smith is a complicated species that is composed of 10 varieties. *Paris polyphylla* Smith var. *yunnanensis* (Franch). Hand.‐Mazz. is one of these varieties, and is mainly distributed in southwestern China (Liang & Soukup, 2000). This variety generally grows under forests and shrubs at an altitude of 1,400–3,100 m (Yang, Li, et al., 2012). Moreover, *P. polyphylla* var. *yunnanensis* is the main plant used in the original formulation of Paridis Rhizoma, a famous traditional Chinese medicine that is used for detoxification and pain relief (Chinese Pharmacopoeia Committee, 2015). This medicine is a major raw material included in multiple Chinese patent medicines, including Gongxuening capsules and Yunnan Baiyao. Due to the high sales of the aforementioned Chinese patent medicines, the demand for Paridis Rhizoma increased by 20% per year, which resulted in the overcollection of wild resources because there was no effective supply available from cultivation (Yang, Li, et al., 2012). In consideration of the endangered status of its natural populations, this variety has been listed as a national key protected wild plant of the second class in China (Huang, Xiao, & Wang, 2011). Since the 1980s, *P. polyphylla* var. *yunnanensis* has been transplanted from wild habitats, and its artificial cultivation gradually became an important way to satisfy the demand for medication derived from this plant (Yang, Li, et al., 2012). According to a recent report, the total cultivated area of this variety was about 2 km^2^ in Yunnan Province, China, but the cultivated supply was still seriously insufficient to meet market demands because of this plant's long growing period (Li, 2016). Furthermore, in field surveys we found that most of the cultivated populations came from provenances with two origins, namely the wild populations of nearby mountains in Yunnan and other cultivation bases. The provenances for cultivated populations are not currently screened, and might include other species or varieties easily confused with this one. Revealing the genetic diversity and structure of *P. polyphylla* var. *yunnanensis* populations would be beneficial to its effective conservation, as well as the breeding of superior provenances for its large‐scale cultivation.

Since it is an important medicinal plant, ISSR, AFLP, and SSR markers have previously been adopted to investigate the genetic diversity and structure of wild populations of *P. polyphylla* var. *yunnanensis* (He, Wang, Li, & Chen, 2007; Li et al., 2018; Song et al., 2015). These studies showed that this variety possessed relatively high genetic diversity and low genetic differentiation among populations. However, the majority of these studies focused on wild populations, and their sample sizes were extremely limited, so they might have been unable to accurately capture the true genetic variation and population structure of *P. polyphylla* var. *yunnanensis*. Although He et al. (2007) compared the genetic characteristics of wild and cultivated populations, only six populations were collected from Yunnan Province, and no populations from other parts of the variety's distribution were included. Therefore, the samples examined in previous studies likely provided relatively poor representations of the variety's distribution, so they could not reveal whether genetic differences existed between wild and cultivated populations of *P. polyphylla* var. *yunnanensis*.

In our study, we collected *P. polyphylla* var. *yunnanensis* from 32 populations within its main distributional range in China, including 15 wild and 17 cultivated populations. We then employed AFLP markers to explore (a) spatial patterns in genetic variation within and among both wild and cultivated populations; (b) genetic differences between the wild and the cultivated populations; and (c) the mixed provenances on which cultivated populations were based.

## 2 MATERIALS AND METHODS

### 2.1 Collection of samples from wild and cultivated populations of *P. polyphylla* var. *yunnanensis*

In this study, a total of 413 individuals of *P. polyphylla* var. *yunnanensis* were sampled from 32 populations, including 15 wild and 17 cultivated populations, from throughout this variety's main geographic distribution in China (Table 1 and Figures S1 and S2). Fresh and healthy leaves were collected from mature individuals for DNA extraction, and dried using silica gel as soon after collection as possible. To avoid repeated sampling from the same female parent, the minimum distance among individuals sampled was 15 m. Over the course of conducting fieldwork, we found that it was extremely difficult to collect enough individuals to satisfy the theoretical requirements for population genetics (generally 20–30 individuals per population), so the numbers of samples collected in some populations were <10. All specimens were identified by Professor Nong Zhou in Chongqing Three Gorges University and then deposited at the Herbarium of Medicinal Plants and Crude Drugs of the College of Pharmacy and Chemistry, Dali University, Dali, China.

**Table 1:** Collecting information on 15 wild and 17 cultivated populations of *P. polyphylla* var. *yunnanensis*

Population code	Locality	Latitude (N)/longitude (E)	Altitude (m)	Sample size
Wild populations				
W‐LL	Longli, Qiannan, Guizhou	26°27′47″N, 106°59′33″E	1,080	6
W‐XY	Qishe, Xingyi, Guizhou	25°0′40″N, 104°49′10″E	1,753	4
W‐HD	Huidong, Xichang, Sichuan	26°23′32″N, 102°57′55″E	2,205	18
W‐YM	Yimen, Yuxi, Yunnan	24°58′38″N, 102°12′52″E	1,893	7
W‐CX	Lvhe, Chuxiong, Yunnan	25°07′55″N, 101°22′30″E	1,871	10
W‐LY	Longyang, Baoshan, Yunnan	25°02′09″N, 099°04′108″E	2,298	11
W‐CN	Changning, Baoshan, Yunnan	24°94′20″N, 099°56′52″E	2,074	9
W‐YP	Yongping, Dali, Yunnan	25°21′27″N, 099°23′14″E	1,925	10
W‐YL	Yunlong, Dali, Yunnan	25°34′57″N, 099°07′29″E	2,268	16
W‐LJ	Yulong, Lijiang, Yunnan	27°01′94″N, 100°22′01″E	3,200	7
W‐LG	Longgong, Anshun, Guizhou	26°5′42″N, 105°52′43″E	1,178	13
W‐XX	Xixiu, Anshun, Guizhou	26°15′50″N, 106°0′35″E	1,410	17
W‐QZ	Weicheng, Qingzhen, Guizhou	26°44′43″N, 106°22′57″E	1,363	10
W‐XR	Xingren, Xingyi, Guizhou	25°32′46″N, 105°27′35″E	1,515	12
W‐GD	Guandu, Kunming, Yunnan	24°59′19″N, 102°58′43″E	2,308	7
Cultivated populations				
C‐LL	Longli, Qiannan, Guizhou	26°27′47″N, 106°59′33″E	1,080	20
C‐XY	Qishe, Xingyi, Guizhou	25°0′40″N, 104°49′10″ E	1,753	5
C‐HD	Huidong, Xichang, Sichuan	26°23′43″N, 102°58′06″ E	2,073	20
C‐YM	Yimen, Yuxi, Yunnan	24°58′38″N, 102°12′52″E	1,893	13
C‐CX	Lvhe, Chuxiong, Yunnan	25°07′55″N, 101°22′30″E	1,871	14
C‐LY	Longyang, Baoshan, Yunnan	25°02′09″N, 099°04′08″E	2,298	20
C‐CN	Changning, Baoshan, Yunnan	24°94′20″N, 099°56′52″E	2,074	20
C‐YP	Yongping, Dali, Yunnan	25°21′08″N, 099°23′06″E	1,829	15
C‐YL	Yunlong, Dali, Yunnan	25°34′57″N, 099°07′29″E	2,172	13
C‐LJ	Yulong, Lijiang, Yunnan	27°01′94″N, 100°22′01″E	3,200	16
C‐ZJ	Zhijin, Anshun, Guizhou	26°48′33″N, 105°38′57″E	1,369	5
C‐ZY	Ziyun, Anshun, Guizhou	25°59′1″N, 106°4′53″E	1,148	5
C‐SM	Songming, Kunming, Yunnan	25°09′25″N, 103°02′31″E	1,996	20
C‐BC	Baicai, Baoshan, Yunnan	25°11′50″N, 099°19′15″E	2,321	15
C‐LX	Longling, Baoshan, Yunnan	24°32′12″N, 098°46′54″E	1,842	20
C‐MS	Mangshi, Dehong, Yunnan	24°29′24″N, 098°20′11″E	1,936	15
C‐JC	Jianchuan, Dali, Yunnan	26°48′42″N, 099°80′75″E	2,944	10

### 2.2 DNA extraction and amplified fragment length polymorphisms (AFLPs)

Total genomic DNA was extracted from about 0.5 g of leaf tissue from each individual plant using the CTAB protocol (Doyle, 1987), with some modifications. The quality of the extracted DNA was checked on 2.0% agarose gels. The original total genomic DNA sample was diluted to 20–50 ng/μl for subsequent AFLP experiments, and stored in a refrigerator at −20°C until further use.

Amplified fragment length polymorphism analysis was performed as described below, according to the standard protocol (Vos et al., 1995), with a few modifications. First, the extracted DNA (200 ng) was digested at 37°C for 3 hr using two restriction enzymes (10 U/μl *Eco*RI and 10 U/μl *Mse*I) in a 20 μl reaction mixture, after which it was kept at 65°C for 3 hr to ensure enzyme inactivation. Next, the digested products were ligated to 50 μM *Eco*RI and 50 μM *Mse*I adaptors using 5 U T4 DNA ligase. Then, 2 μl of the ligation product was pre‐amplified with 0.5 μl of each single selective primer (20 μM E00 and 20 μM M00), 2 μl of 10 × T4 buffer, and 0.4 μl of 10 mM dNTPs in a final volume of 20 μl. Amplification conditions were the following: 3 min at 94°C; 45 s at 94°C; and 26 cycles of 45 s at 50°C and 1 min at 72°C. The pre‐amplified product was then diluted 20‐fold, and 2 μl of the diluted product was selectively amplified with 2 μl of 10 × buffer, 0.4 μl of 10 mM dNTPs, 0.2 μl of 5 U Taq, 1 μl of 20 μM EcoRI primer, and 1 μl of 20 μM MseI primer (E37A‐CGA/M51A‐CAA, E37A‐CGA/M52G‐CCG, E37A‐CGA/M51T‐CAT, E85T‐CGT/M52G‐CCG, E40‐AGC/M51T‐CAT) in a final volume of 20 μl. The thermal cycling parameters for this selective amplification were as follows: 5 min at 95°C; 12 cycles of 35 s at 95°C, 35 s at 65°C (with annealing initiated at a temperature of 65°C, which was then reduced for each of 12 subsequent cycles by 0.7°C), 1 min at 72°C; and 23 subsequent cycles of 30 s at 94°C, 30 s at 56°C, and 1 min at 72°C. Finally, the selective amplification products were 10‐fold diluted and mixed with the GeneScan 500 LIZ size standard, and then, the mixed products were detected using an ABI 3730XL automated DNA sequencer (Applied Biosystems™). In fragment analysis, FAM was adopted to label primers of AFLP markers. Fragments were analyzed with GeneMarker v.1.70 (Soft Genetics, LLS). Fragments were scored as either present (1) or absent (0) when the intensity was up to 100.

### 2.3 Data analysis

Genetic diversity parameters were calculated using PopGen32 (Yeh, Yang, & Boyle, 1999), including the effective number of alleles (*N*_E_), Nei's genetic diversity (*H*), Shannon's information index (*I*), and the percentage of polymorphic bands (PPB). ArcGIS v. 10.3 (Esri) was used to draw maps of the geographic distribution of the sampled wild and cultivated populations of *P. polyphylla* var. *yunnanensis*.

PopGen32 was used to construct an unweighted pair group method with arithmetic mean (UPGMA) dendrogram (Michener & Sokal, 1957) at the population level for all of the populations sampled, including both the wild and the cultivated populations. In addition, NTSYS‐pc v. 2.1 (Rholf, 2000) was used to construct UPGMA dendrograms for the populations in Yunnan at the individual level to explore the probable genetic promiscuity of the bases of these cultivated populations. Pairwise distances among individuals were calculated in terms of Jaccard's coefficient values (Jaccard, 1908), and then, a matrix of Jaccard distances was employed to cluster the individual samples.

STRUCTURE v. 2.3.4 was also used to infer the genetic structure of the 32 wild and cultivated populations (Falush, Stephens, & Pritchard, 2007; Pritchard, Stephens, & Donnelly, 2000). To predict the optimum number of clusters (*K*), an admixture model and correlated allele frequencies were chosen for use in this analysis. At the same time, ten replicates of each simulation with 2–10 clusters (*K* = 2–10) were simulated through 10,000 Markov chain Monte Carlo (MCMC) steps by sampling after a burn‐in period of 10,000 iterations. STRUCTURE HARVESTER was used to extract the relevant data from the structure results files and to generate CLUMPP input files (Earl & vonHoldt, 2011). Replicates were then built with CLUMPP v. 1.1.2, using the FullSearch option to determine the optimal *K* value. The membership of different cluster groups was visualized using DISTRUCT v. 1.1 (Jakobsson & Rosenberg, 2007; Rosenberg, 2003).

GenAlEx v. 6.503 was used to perform a principal coordinates analysis (PCoA) to reveal the relationships among populations (Peakall & Smouse, 2012), as well as a Mantel test to assess the correlation between the geographic distance (in km) and the genetic distance among different wild and cultivated populations, and also among only the cultivated populations in Yunnan (Mantel, 1967). To be comparable with the UPGMA results, PCoA was similarly performed for all of the sampled populations, including both the wild and the cultivated populations together, as well as separately. Analysis of molecular variance (AMOVA) was performed to quantify the genetic variation at different hierarchical levels with 1,000 permutations using Arlequin version 3.5 (Excoffier & Lischer, 2010). Both cases with grouping and no grouping of populations were considered in the AMOVA.

## 3 RESULTS

### 3.1 Genetic diversity within wild and cultivated populations

In this study, a total of 669 loci were detected by five of the selected pairs of AFLP primers. Assuming Hardy–Weinberg equilibrium, Nei's genetic diversity (*H*), an important parameter for evaluating genetic diversity, was higher in the cultivated populations than that in the wild ones at the species level (*H* = 0.2636 vs. 0.2616, respectively), whereas the value was as high as 0.2657 if all 32 of the sampled populations were considered together (Table 2). Meanwhile, the mean value of *H* for the cultivated populations at the population level was also higher than the mean value for the wild populations (*H* = 0.2002 vs. 0.2001, respectively).

**Table 2:** Genetic diversity of *P. polyphylla* var. *yunnanensis* based on AFLP marker

Populations	*N*	*N*_E_	*H*	*I*	NPB	PPB
Subgroup W‐I						
W‐LL	6	1.3614 (0.0142)	0.2141 (0.0075)	0.3232 (0.0107)	420	62.78%
W‐XY	4	1.3092 (0.0144)	0.1809 (0.0076)	0.2710 (0.0110)	337	50.37%
W‐LG	13	1.3969 (0.0143)	0.2337 (0.0075)	0.3537 (0.0104)	485	72.50%
W‐XX	17	1.4059 (0.0139)	0.2419 (0.0072)	0.3683 (0.0100)	522	78.03%
W‐QZ	10	1.3700 (0.0144)	0.2171 (0.0076)	0.3275 (0.0107)	440	65.77%
W‐XR	12	1.3673 (0.0137)	0.2211 (0.0072)	0.3393 (0.0100)	486	72.65%
Mean	10.3	1.3685 (0.0058)	0.2181 (0.0031)	0.3305 (0.0043)	448.4	67.02%
Subgroup W‐I level	62	1.4220 (0.3417)	0.2555 (0.1743)	0.3941 (0.2342)	619	92.53%
Subgroup W‐II						
W‐YM	7	1.3089 (0.0141)	0.1823 (0.0076)	0.2755 (0.0108)	364	54.41%
W‐CX	10	1.3362 (0.0143)	0.1982 (0.0076)	0.2999 (0.0108)	406	60.69%
W‐GD	7	1.3201 (0.0142)	0.1887 (0.0076)	0.2851 (0.0108)	377	56.35%
Mean	8.0	1.3218 (0.0082)	0.1897 (0.0044)	0.2868 (0.0062)	382.3	57.15%
Subgroup W‐II level	24	1.3541 (0.3462)	0.2156 (0.1826)	0.3332 (0.2557)	505	75.49%
Subgroup W‐III						
W‐HD	18	1.4158 (0.0139)	0.2474 (0.0071)	0.3783 (0.0097)	555	82.96%
W‐LY	11	1.2782 (0.0137)	0.1656 (0.0074)	0.2531 (0.0105)	363	54.26%
W‐CN	9	1.2868 (0.0144)	0.1665 (0.0076)	0.2509 (0.0109)	338	50.52%
W‐YP	10	1.2761 (0.0136)	0.1645 (0.0074)	0.2505 (0.0106)	347	51.87%
W‐YL	16	1.3445 (0.0141)	0.2053 (0.0074)	0.3157 (0.0103)	476	71.15%
W‐LJ	7	1.3032 (0.0145)	0.1759 (0.0077)	0.2642 (0.0110)	345	51.57%
Mean	11.8	1.3174 (0.0058)	0.1875 (0.0031)	0.2855 (0.0043)	404.0	60.39%
Subgroup W‐III level	71	1.3905 (0.3537)	0.2359 (0.1794)	0.3664 (0.2416)	605	90.43%
Wild mean	10.5	1.3387 (0.0037)	0.2001 (0.0019)	0.3037 (0.0028)	417.4	62.39%
Wild level	157	1.4353 (0.3400)	0.2616 (0.1703)	0.4072 (0.2250)	652	97.46%
Subgroup C‐I						
C‐LL	20	1.3020 (0.0137)	0.1804 (0.0074)	0.2751 (0.0106)	398	59.49%
C‐XY	5	1.2207 (0.0134)	0.1285 (0.0072)	0.1922 (0.0105)	241	36.02%
C‐ZJ	5	1.2264 (0.0137)	0.1299 (0.0074)	0.1926 (0.0106)	234	34.98%
C‐ZY	5	1.2311 (0.0137)	0.1333 (0.0074)	0.1985 (0.0106)	246	36.77%
Mean	8.8	1.2451 (0.0068)	0.1430 (0.0037)	0.2146 (0.0053)	279.8	41.82%
Subgroup C‐I level	35	1.3142 (0.3579)	0.1879 (0.1907)	0.2888 (0.2692)	453	67.71%
Subgroup C‐II						
C‐YM	13	1.4040 (0.0145)	0.2366 (0.0075)	0.3571 (0.0105)	487	72.80%
C‐CN	20	1.4137 (0.0139)	0.2461 (0.0072)	0.3745 (0.0099)	530	79.22%
C‐YP	20	1.4341 (0.0140)	0.2567 (0.0072)	0.3894 (0.0098)	546	81.61%
C‐LJ	16	1.4191 (0.0142)	0.2470 (0.0073)	0.3740 (0.0101)	522	78.03%
C‐SM	20	1.4543 (0.0139)	0.2686 (0.0070)	0.4076 (0.0095)	572	85,50%
C‐MS	15	1.4192 (0.0137)	0.2504 (0.0071)	0.3820 (0.0097	540	80.72%
Mean	17.3	1.4241 (0.0057)	0.2509 (0.0029)	0.3808 (0.0041)	532.9	79.65%
Subgroup C‐II level	104	1.4741 (0.3397)	0.2838 (0.1668)	0.4342 (0.2181)	649	97.01%
Subgroup C‐III						
C‐HD	20	1.3539 (0.0138)	0.2132 (0.0072)	0.3294 (0.0100)	511	76.38%
C‐CX	14	1.2961 (0.0137)	0.1765 (0.0074)	0.2688 (0.0106)	377	56.35%
C‐LY	20	1.3426 (0.0136)	0.2078 (0.0071)	0.3223 (0.0099)	503	75.19%
C‐YL	13	1.2949 (0.0136)	0.1764 (0.0074)	0.2693 (0.0106)	383	57.25%
C‐BC	15	1.3129 (0.0139)	0.1870 (0.0074)	0.2872 (0.0104)	427	63.83%
C‐LX	20	1.2818 (0.0131)	0.1713 (0.0072)	0.2644 (0.0103)	401	59.94%
C‐JC	15	1.3230 (0.0140)	0.1924 (0.0074)	0.2941 (0.0105)	425	63.53%
Mean	16.7	1.3150 (0.0052)	0.1892 (0.0028)	0.2908 (0.0039)	432.4	64.64%
Subgroup C‐III level	117	1.3604 (0.3508)	0.2193 (0.1798)	0.3432 (0.2438)	607	90.73%
Cultivated mean	15.1	1.3371 (0.0034)	0.2002 (0.0018)	0.3046 (0.0026)	432.0	64.57%
Cultivated level	256	1.4333 (0.3452)	0.2636 (0.1717)	0.4046 (0.2255)	660	98.65%
W&C mean	12.9	1.3378 (0.0025)	0.2002 (0.0013)	0.3042 (0.0019)	425.1	63.55%
W&C level	413	1.4393 (0.3414)	0.2657 (0.1693)	0.4109 (0.2214)	667	99.70%

Note

*H*, Nei's genetic diversity; *I*, Shannon's information index; *N*, number of materials; *N*_E_, effective number of alleles; NPB, number of polymorphic bands; PPB, percentage of polymorphic bands.

Among the wild populations, the HD population from along the southern margin of Sichuan Province showed the highest genetic diversity (0.2474). Moreover, most of the populations in Guizhou Province possessed relatively high genetic diversity values, except for XY, from which only four individuals were sampled. On the other hand, the wild populations in Yunnan showed \lower genetic variation than the others, especially the YP, LY, and CN populations in western Yunnan. However, among the cultivated populations, the situation was completely different compared with that among the wild ones. The cultivated populations in Guizhou possessed much lower genetic diversity than those in Yunnan. For instance, the LL population in Guizhou, from which 20 individuals were sampled, still showed significantly lower genetic variation than the SM and YP populations in western Yunnan.

**Figure 1:**
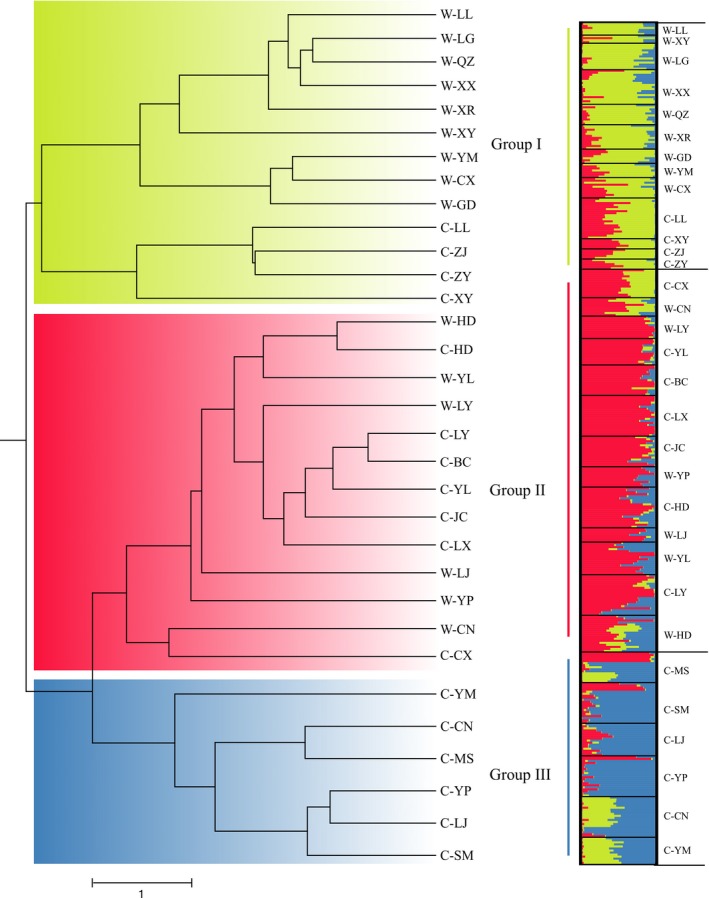
Genetic structure of 32 populations of *P. polyphylla* var. *yunnanensis* estimated by UPGMA dendrogram and STRUCTURE analysis. The populations were divided into three groups according to the UPGMA dendrogram, consistent with results of the STRUCTURE. On the STRUCTURE bar plots, each color stands for one group and black lines isolate populations that were labeled below the figure

**Figure 2:**
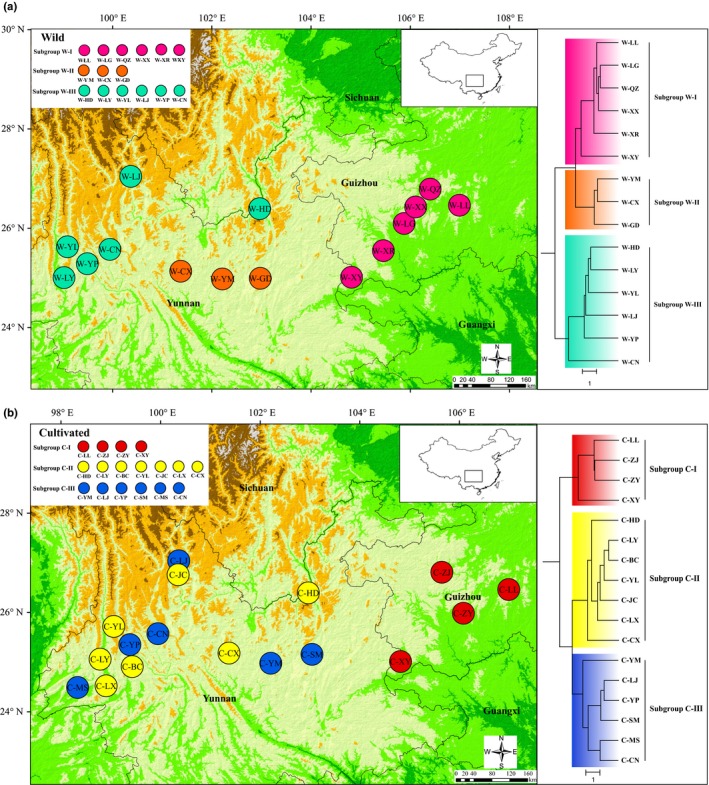
Distributions and UPGMA dendrograms of wild and cultivated populations. (a) Pink, subgroup W‐I; orange, subgroup W‐II; green, subgroup W‐III. (b) Red, subgroup C‐I; yellow, subgroup C‐II; blue, subgroup C‐III

When the groupings of wild populations identified in our clustering analysis were considered (Figures 1 and 2), subgroup W‐I, located in Guizhou, possessed higher genetic diversity at the population level (*H* = 0.2181) than that possessed by the remaining subgroups in Yunnan (*H* = 0.1897 and 0.1875 in W‐II and W‐III, respectively). However, a totally different situation was found for the cultivated populations. The genetic variation within the subgroup C‐I (*H* = 0.1430), also located in Guizhou, was lower than that in the other subgroups in Yunnan (*H* = 0.1892 and 0.2509 in C‐II and C‐III, respectively).

### 3.2 Population structure and genetic differentiation

**Figure 3:**
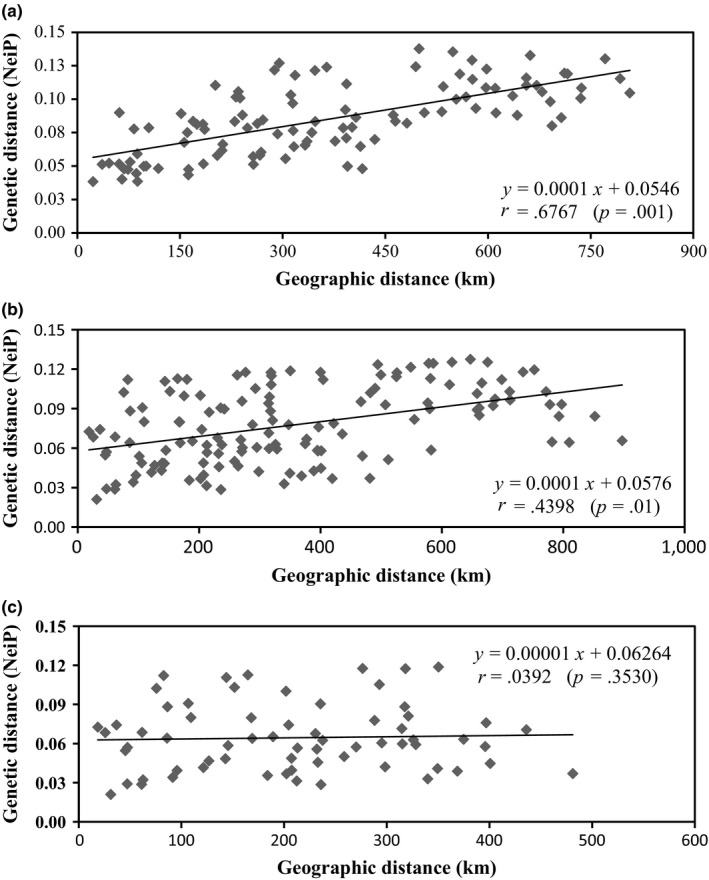
Correlations between geographic distances (km) and genetic distances (NeiP). (a) Mantel test for wild populations. (b) Mantel test for cultivated populations. (c) Mantel test for cultivated populations in Yunnan Province

In the present study, three UPGMA dendrograms were constructed for all 32 populations, the wild populations, and the cultivated populations, respectively, at the population level. In the first of these (Figure 1 and Figure S3), the 32 populations were roughly divided into three main groups (groups I, II, and III). Both the wild and cultivated populations in Guizhou formed group I, but they were clustered into two separate subgroups within this group. The remained populations, mainly those in Yunnan, were clustered into two groups (groups II and III). Group II was relatively complicated, including both wild and cultivated populations, whereas group III was only composed of six cultivated populations. Furthermore, the UPGMA dendrogram for the wild populations only also gathered these into three main subgroups, namely subgroups W‐I, W‐II, and W‐III (Figure 2), which showed obvious genetic structuring that was also supported by STRUCTURE and PCoA results. Meanwhile, the Mantel test indicated there was a significantly positive correlation between the genetic and geographic distances among these populations (*r* = .6767, *p* = .001) (Figure 3). On the contrary, although the final UPGMA dendrogram also divided the 17 cultivated populations into three main subgroups (subgroups C‐I, C‐II, and C‐III), their genetic structuring was obviously not consistent with their geographic distribution, except within subgroup C‐I in Guizhou (Figure 2). The remaining two cultivated subgroups, containing populations located in Yunnan, did not show any correlation between genetic and geographic distances, which was also supported by the Mantel test results (Figure 3). Moreover, the UPGMA dendrogram produced at the individual level based on NTSYS analysis (Figure S4) indicated that individuals cultivated from different bases were clustered into the same group, such as those in the cultivated populations YP and JC.

The most likely number of clusters (*K*) was inferred by the peak Δ*K* found in the STRUCTURE analysis. According to the method of Evanno, Regnaut, and Goudet (2005), the 32 populations were split into *K* = 3 groups (Figure 1), which was consistent with the result of the UPGMA dendrogram. Similarly, the optimal *K* value for the wild and the cultivated populations was also equal to 3, supporting each of the UPGMA dendrograms found above (Figure 2).

**Figure 4:**
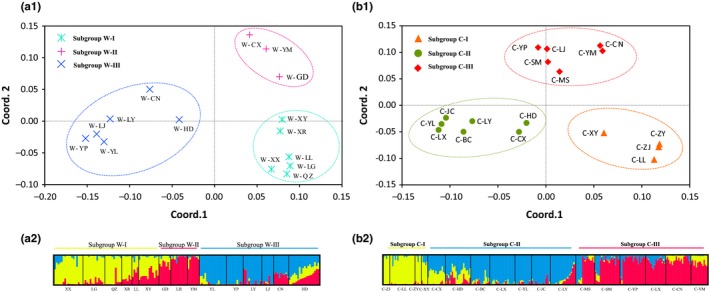
Principal coordinates analysis (PCoA) and Bayesian clustering by STRUCTURE of wild populations and the cultivated. (a1 and b1) A dot line circle represents a subgroup. “*” represents subgroup W‐I; “+” represents subgroup W‐II; “×” represents subgroup W‐III; “▲” represents subgroup C‐I; “●” represents subgroup C‐II; “◆” represents subgroup C‐III. (a2 and b2) Each color represents a subgroup

The PCoA performed on the basis of all 32 populations revealed that the populations that belonged to three different groups according to the UPGMA dendrogram of all populations could not be well separated in this analysis, especially the two groups in Yunnan Province (Figure S3). However, the wild populations were clearly clustered into three subgroups in the PCoA, in accordance with their UPGMA dendrogram, and so were the cultivated populations (Figure 4). Meanwhile, the PCoA results were also supported by the previously mentioned STRUCTURE analysis.

Regardless of the grouping conditions applied, AMOVA results showed that genetic variation was mainly concentrated within populations for all of the sampled populations, the wild populations only, and the cultivated populations only, with low percentages of variance among populations similar Φ_ST_ values found for these, of 19.73, 18.83, and 19.39%, respectively (Table 3). When considering the grouping conditions in the AMOVA, the genetic variation among groups or subgroups was as high as 9.28%, 12.11%, and 12.70% for all populations, wild ones only, and cultivated ones only, respectively, supporting the clear genetic structuring found in the previous analyses. On the contrary, if the wild and cultivated populations were regarded as two separate groups, there was a genetic variation of only 1.35% between these groups, which indicated that there was no significant overall genetic differentiation between the wild and cultivated populations.

**Table 3:** Analysis of molecular variance (AMOVA) for all the populations, the wild and the cultivated of *P. polyphylla* var. *yunnanensis*

Source of variance	*df*	SSD	VC	PV (%)	Fixation indices
All the populations without hierarchy					
Among populations	31	10,578.785	20.18495	19.72507	*F*_ST_ = 0.19725*
Within populations	412	31,297.825	82.14652	80.27493	
Total	443	41,876.610	102.33148		
Wild populations versus cultivated populations					
Among groups	1	623.190	1.38889	1.34790	*F*_CT_ = 0.01348*
Among populations within groups	30	9,955.595	19.50563	18.92996	*F*_SC_ = 0.19189*
Within populations	381	31,297.825	82.14652	79.72214	*F*_ST_ = 0.20278*
Total	412	41,876.610	103.04104		
All the populations with hierarchy (UPGMA)					
Among groups	2	3,180.061	9.79758	9.27932	*F*_CT_ = 0.09279*
Among populations within groups	29	7,398.724	13.64107	12.91949	*F*_SC_ = 0.14241*
Within populations	381	31,297.825	82.14652	77.80119	*F*_ST_ = 0.22199*
Total	412	41,876.610	105.58518		
Wild populations without hierarchy					
Among populations	14	3,967.428	19.32002	18.83066	*F*_ST_ = 0.18831*
Within population	142	11,825.579	83.27872	81.16934	
Total	156	15,793.006	102.59874		
Wild populations with hierarchy					
Among subgroups	2	2,507.832	12.78588	12.11474	*F*_CT_ = 0.12161*
Among populations within subgroups	12	3,480.336	11.28014	10.68804	*F*_SC_ = 0.22803*
Within populations	142	19,472.246	81.47383	77.19722	*F*_ST_ = 0.12115*
Total	156	25,460.414	105.53985		
Cultivated populations without hierarchy					
Among populations	16	5,988.168	19.59684	19.38932	*F*_ST_ = 0.19389*
Within population	239	19,472.246	81.47383	80.61068	
Total	255	25,460.414	101.07077		
Cultivated populations with hierarchy					
Among subgroups	2	1703.202	13.60456	12.69944	*F*_CT_ = 0.12699*
Among populations within subgroups	14	2,264.225	10.24394	9.56240	*F*_SC_ = 0.10953*
Within populations	239	11,825.579	83.27872	77.73816	*F*_ST_ = 0.22262*
Total	255	15,793.006	107.12722		

Note

*df*, degree of freedom; PV, percentage of variance; SSD, sum of squared deviations; VC, variance component.

* Significant difference at the *p* < .001 level.

## 4 DISCUSSION

### 4.1 Genetic diversity of wild populations of *P. polyphylla* var. *yunnanensis*

Due to the impact of genetic drift and inbreeding, species with narrow geographic distributions and smaller‐sized individuals generally show lower genetic diversity than more widespread ones (Li, Guan, Yang, Luo, & Chen, 2012; Willi, Van Buskirk, & Hoffmann, 2006). Therefore, endangered and rare species are regarded as being deficient in genetic variation (Hamrick & Godt, 1989). Seventeen species of *Paris* have been listed as endangered species under the National Protection Grade II classification in China, including *P. polyphylla* var. *yunnanensis* (http://rep.iplant.cn/). During fieldwork, we found that this variety was widely distributed in southwestern China, but it was nearly impossible to collect enough individuals for sufficient statistical power (*n* = 30) from most of its wild populations. Therefore, the evaluation of the genetic diversity of wild populations of this variety should be vital for the formulation of protection strategies and the utilization of wild resources. The present study revealed that the genetic diversity of *P. polyphylla* var. *yunnanensis* at the species level (*H* = 0.2636) (Table 2) was similar to that previously reported for this species (*H* = 0.2768 for AFLPs) (Li et al., 2018), as well as its relatives, such as *Paris thibetica* var. *apetala* (*H* = 0.2849) and *P. thibetica* var. *thibetica* (*H* = 0.2439) (Tang, Wang, & Weng, 2008). Although He et al. (2007) detected lower genetic diversity than this in three wild populations of *P. polyphylla* var. *yunnanensis* (*H* = 0.187) based on the analysis of ISSR markers, this difference in the diversity detected could be attributed to differences in sample sizes and marker types among studies (Zhang & Yang, 2008). Moreover, the present study also indicated that the genetic diversity of this variety was higher (Table 2) than that of several other endangered species assessed based on AFLP markers, such as *Lysimachia tashiroi* (*H* = 0.1540) (Nakazawa & Yahara, 2007), *Sinopodophyllum hexandrum* (*H* = 0.035) (Li et al., 2011), and *Artemisia pancicii* (*H* = 0.036) (Kitner, Majeský, Gillová, Vymyslický, & Nagler, 2012), but lower than that of *Fritillaria cirrhosa* D. Don (*H* = 0.3709) (Zhang, Gao, & Yang, 2010). These comparisons indicate that the endangered status of the wild populations of *P. polyphylla* var. *yunnanensis* can likely be attributed to overcollecting and human disturbance, rather than to their biological characteristics.

In this study, we mainly collected samples from wild populations in Yunnan and Guizhou, as well as one population in Sichuan (Table 1). Our findings revealed that the wild populations in Guizhou possessed high genetic diversity at the population level, such as the XX, LG, and XR populations (subgroup W‐I). Conversely, the wild populations in Yunnan showed low genetic variation, especially YP, LY, and CN in western Yunnan (subgroup W‐III). A series of Chinese patent medicines (e.g., Yunnan Baiyao) require the use of massive amounts of medicinal materials of Paridis Rhizoma, so cultivation of *P. polyphylla* var. *yunnanensis* has been popular in Yunnan over the last decade (Yang, Li, et al., 2012). However, the cultivation of this variety might result in the destruction of its habitats and wild populations if a large number of wild individuals are collected and used for seedlings in cultivation. We deduced that this might be an important reason for the low genetic variation in Yunnan populations revealed in the present study. It should be noted that the HD population located along the southern margin of Sichuan showed the highest diversity of the 15 sampled wild populations (*H* = 0.2474). It was regrettable that samples were only collected from one population (HD) in Sichuan, so further surveys and studies on the populations of this variety distributed in Sichuan and adjacent regions will be extremely important. The wild populations in Guizhou and Sichuan should thus be regarded as priorities for protection, and used for the screening of superior provenances for future cultivation efforts.

### 4.2 Genetic diversity of cultivated populations and comparisons between wild and cultivated populations

Generally speaking, wild populations have higher genetic diversity than cultivated ones because of the bottleneck effects, founder effects, and genetic drift that occur during the process of demonstration and cultivation (Miller & Schaal, 2006; Wu, Li, & Huang, 2006). However, in this study, the genetic variation in cultivated populations was found to be higher than that in the wild ones at the species level (*H* = 0.2636 vs. 0.2616, respectively), as well as at the population level (*H* = 0.2002 vs. 0.2001), indicating that cultivation of *P. polyphylla* var. *yunnanensis* has not yet resulted in any obvious loss of genetic variation. Our findings were consistent with those reported in a previous study (He et al., 2007). These results indicated that the seedlings of *P. polyphylla* var. *yunnanensis* currently used as the bases of most cultivation efforts have complicated genetic backgrounds; in other words, the individuals cultivated in the same place might have come from multiple wild source populations, or even different genetic lineages (He et al., 2007). This conclusion was also supported by our NTSYS analysis of the cultivated individuals in Yunnan (Figure S4). The high genetic diversity found in the cultivated populations showed that the genetic resources of *P. polyphylla* var. *yunnanensis* could gain effective protection through cultivation, but did not satisfy the requirements for producing uniform and high‐quality material for Paridis Rhizoma. Therefore, the next task for the cultivation of *P. polyphylla* var. *yunnanensis* should be screening and fostering superior cultivated varieties from the currently complicated, but rich, genetic resources available.

### 4.3 Spatial genetic structure of wild and the cultivated populations

The spatial genetic structure of a species' populations reflects the interactions involved in their long‐term evolutionary history, such as habitat fragmentation, genetic drift, type of mating system, and gene flow (Schaal, Haryworth, Olsen, Auscher, & Smith, 1998). In this study, the 15 wild populations sampled were divided into three subgroups (W‐I, W‐II, and W‐III) based on the UPGMA dendrogram produced, which corresponded to an obvious geographic structure (Figure 2) that was in accordance with the results of PCoA and STRUCTURE (Figure 4). Moreover, this geographic structure was also supported by the results of the Mantel test, which revealed that there was a significant, positive correlation between the genetic and geographic distances among populations (Figure 3). The W‐I subgroup was composed of six populations located in Guizhou Province; meanwhile, the remaining two subgroups were comprised of populations that were mainly distributed in Yunnan Province, namely the central (W‐II) and western (W‐III) parts of the province. It was surprising that the W‐I subgroup showed a closer relationship with the W‐II subgroup than the W‐III subgroup did, despite the shorter geographic distances between the W‐II and W‐III populations (Figure 2). These relationships might have been caused by different terrain features in these parts of the species' distribution. It is well known that the Yunnan–Guizhou Plateau, as the second highest steppe area in Chinese topography, is composed of two plateaus without clear boundaries, namely Yunnan Plateau and Guizhou Plateau (Chen & Wang, 2017). Meanwhile, the topography of Yunnan Province is roughly divided into two parts by the Yuan River: the eastern region belongs to the Yunnan–Guizhou Plateau, but the classification of the western region, namely western Yunnan, is controversial, and it is sometimes ascribed to either the Qing‐Tibetan Plateau or the Yunnan–Guizhou Plateau (Chen & Wang, 2017; Wang, Yang, He, & Wang, 2012). In the present study, the wild populations in the W‐I and W‐II subgroups were all distributed on the Yunnan–Guizhou Plateau, whereas those in the W‐III subgroup were located on the western Yunnan Plateau or along the southern margins of the Hengduan Mountains. Among the three subgroups, W‐I showed the highest genetic diversity, with an average *H* of 0.2181 at the population level, whereas that of the W‐II (0.1897) and W‐III (0.1875) subgroups was lower, suggesting that the populations on the Guizhou Plateau might be more valuable than others for wild conservation and the breeding of cultivated varieties of *P. polyphylla* var. *yunnanensis*.

According to the UPGMA dendrogram of the cultivated populations (Figure 2), the 17 cultivated populations were also clustered into three independent subgroups (C‐I, C‐II, and C‐III), which were supported by the results of STRUCTURE and PCoA (Figure 4). When considering the populations' geographic distributions (Figure 2), there was significant geographic structuring between the populations in Guizhou (C‐I) and those in Yunnan (C‐II and C‐III), which was explained by the Mantel test finding an obvious and significant positive correlation between the genetic and geographic distances between them (*r* = .4398, *p* < .01) (Figure 3). However, the Mantel test done for the cultivated populations located in Yunnan Province indicated that no significant correlation existed between these two types of distances between the populations in subgroups C‐II and C‐III (*r* = .0387, *p* > .05) (Figure 3). The current spatial genetic structure of the cultivated populations in Yunnan Province could be attributed to three possible reasons. First, from the UPGMA dendrogram created at the individual level (Figure S4), some individuals from the same cultivation base were clustered into multiple clades, but other individuals from some different bases also formed one clade together. This result indicated that the provenances of *P. polyphylla* var. *yunnanensis* in many cultivated populations were mixed, and thus, they might have been derived from multiple source populations; indeed, the mutual introduction of provenances from different sources among cultivated populations was previously popular in Yunnan. Second, *P*. *polyphylla* is an extremely complicated species that is composed of 10 varieties, including *P. polyphylla* var. *yunnanensis* (Liang & Soukup, 2000). Technicians working on cultivated populations are generally unable to discriminate the subtle differences in morphology among varieties due to a lack of taxonomic knowledge, and thus, they might introduce incorrect provenances, resulting in the production of cultivated lines with mixed provenances (Figure S4). Finally, compared with other well‐demonstrated crops, *P. polyphylla* var. *yunnanensis* is still in the early stages of demonstration and cultivation, so it has not yet experienced a long history of selection and breeding (Brown, 1978; Ellstrand & Marshall, 1985; Hamrick & Godt, 1997; Hyten et al., 2006; Wang et al., 2017).

During fieldwork, we found that the cultivation of *P. polyphylla* var. *yunnanensis* was much more popular in Yunnan than in Guizhou, which was also reflected in our collection information (Table 1). According to the UPGMA dendrogram of all the populations (Figure 1), cultivated populations in Yunnan were almost never based on the introduction of provenances from Guizhou, and *vice versa*. Considering the rich genetic diversity of the wild populations in Guizhou, it is necessary to utilize the wild resources there for the selection and breeding of new cultivated varieties. For the cultivated populations in Yunnan Province, there were two obvious subgroups of different provenances (C‐II and C‐III). Among these, the populations in the C‐II subgroup were mixed with the wild populations (Figure 1), whereas the populations in the C‐III subgroup were obviously separated from them, indicating that the latter might have experienced a longer history of domestication. It is also worth noting that most cultivation bases operating beyond a certain scale generally introduce provenances from other bases rather than collecting seedlings from wild habitats.

According to the AMOVA results, there was no obvious overall genetic differentiation between the sampled wild and cultivated populations, as only 1.35% of the genetic variation found existed between these two types of populations (Table 3). Moreover, without grouping included in the AMOVA, the wild populations of *P. polyphylla* var. *yunnanensis* showed relatively low genetic differentiation (Φ_ST_ = 18.83%) compared with that detected in previous studies of *F. cirrhosa* D. Don (Zhang et al., 2010) and *Camellia reticulata* (Xin et al., 2017), as well as statistical data (Nybom, 2004). This revealed that enough gene flow among wild populations might have occurred to have effectively prevented the aggravating of among‐population genetic differentiation. Although a similar Φ_ST_ value was detected in the cultivated populations (19.39%), the low genetic differentiation among these should likely be attributed to the mutual introduction of some of the same provenances among the bases of cultivated populations. Moreover, the spatial genetic structure of the wild populations was relatively significant based on the UPGMA dendrogram, STRUCTURE analysis, and PCoA results, but since only 12.11% of the genetic variation found existed among groups, this indicated that the spatial structuring of these populations might not be very strong.

### 4.4 Conservation

Demand for traditional medicines is extremely high all over the world, and is still increasing (Hamilton, 2004). The supply of Paridis Rhizoma, an important herbal medicine, cannot currently meet this increasing demand of 20% each year (Yang, Li, et al., 2012; Yang et al., 2012) because the growth of the original plants from which it is made, namely *P. polyphylla* var. *yunnanensis*, is very slow; in fact, it takes more than 10 years for wild individuals to develop from the seed germination stage to harvest under natural growth conditions (Yang, Li, et al., 2012; Yang, Yang, et al., 2012). Artificial cultivation should be an effective method to protect the wild resources of this plant and guarantee their sustainable utilization (Allendorf & Luikart, 2007; Storfer, 1999). It was previously reported that in traditional cultivation methods, farmers collected seeds directly from wild populations and then cultivated them in similar habitats, which was a useful way to maintain the gene pools of these medicinal plants (Guo et al., 2007; He et al., 2009). Based on our study's results, this traditional manner of cultivation was only enacted in minor, remote mountain areas, where it might have maintained rare loci from the local populations, such as the HD population from southern Sichuan. On the contrary, most of the bases of cultivated populations, especially the large ones, generally introduced seedlings obtained from mountain villagers or other cultivated populations. Although these populations showed high genetic variation in our study, their confusing provenances might result in them producing Paridis Rhizoma of heterogeneous quality. Therefore, in this context it is impossible to maintain individuals belonging to different specific genotypes in cultivated populations in the long run. In our opinion, germplasm banks and medicinal botanical gardens established for ex situ conservation should play major roles in the conservation of genetic diversity and rare genes from different populations. Meanwhile, varieties with good quality should be bred, which could then form the bases of new cultivated lines.

Although both artificial cultivation and ex situ conservation are effective means of protecting *P. polyphylla* var. *yunnanensis*, the in situ conservation of wild populations should still be the fundamental method applied. Generally speaking, wild populations with richer genetic variation possess higher conservation value (Soldati, Fornes, Zonneveld, Thomas, & Zelener, 2013; Yao, Deng, & Huang, 2012). In the present study, the populations located in Guizhou Province showed higher genetic diversity compared with those in Yunnan, leading us to propose that the wild populations in Guizhou should be given priority protection and subjected to restricted harvest rules. Meanwhile, the HD population in Sichuan also possessed rich genetic variation at the population level (*H* = 0.2474), so southern Sichuan should be also be listed as a priority site for further study. Furthermore, reasonable manual intervention, such as removing seed coats and then sowing seeds in wild habitats, could improve the germination rates of seeds, and thus would benefit the renewal and maintenance of effective wild populations.

## 5 CONCLUSIONS

In this study, AFLP markers were adopted as a means to explore and compare the genetic diversity and population structure of wild and cultivated populations of *P. polyphylla* var. *yunnanensis*. The result revealed that there was moderate genetic variation among wild populations. Wild populations located in Guizhou Province possessed higher genetic diversity than populations in Yunnan Province, suggesting that populations in Guizhou should be given priority protection. Compared with wild populations, the cultivated ones did not show obviously lower genetic variation. However, further analyses at the individual level revealed the confusing provenances of the bases of many cultivated populations. UPGMA dendrograms divided the wild populations into three subgroups (W‐I, W‐II, and W‐III) with a clear spatial geographic structure, which was also supported by STRUCTURE, Mantel test, and PCoA results. However, although the cultivated populations were also clustered into three subgroups (C‐I, C‐II, and C‐III), the latter two subgroups, which were mainly located in Yunnan, did not show significant correlations between the genetic and geographic distances among populations within them. This study indicated that cultivated seedlings had complicated and confusing origins, so the selection and breeding of varieties with good and homogenous quality should be listed as a priority for the future cultivation of *P. polyphylla* var. *yunnanensis*. This study not only explored the genetic diversity and population structure of wild populations of *P. polyphylla* var. *yunnanensis*, but also revealed that cultivated populations presented some defective provenances. These findings could be beneficial to both the conservation of wild resources and the development of more robust cultivation approaches for this medicinal species.

## CONFLICT OF INTEREST

The authors declare that they have no conflict of interest.

## AUTHOR CONTRIBUTIONS

Y.H. collected samples, analyzed the data, and wrote the primary manuscript. N.Z. performed fieldwork and covered the costs of this study. M.Y. performed the main experiments, and Y.X.S. helped to perform field investigations. D.Q.Z. conceived the research, analyzed the data, and revised the final manuscript.

## Data Availability

The raw data of AFLP markers used in the present study are available through the figshare database (https://doi.org/10.6084/m9.figshare.9248483.v1).
