# Supplementary figures and images for: A comparative study of the population genetics of wild and cultivated populations of Paris polyphylla var. yunnanensis based on amplified fragment length polymorphism markers
Source: Ecol Evol. 2019 Aug 20;9(18):10707–22. doi: 10.1002/ece3.5589 (PMC6787796; doi:10.1002/ece3.5589)

(A)

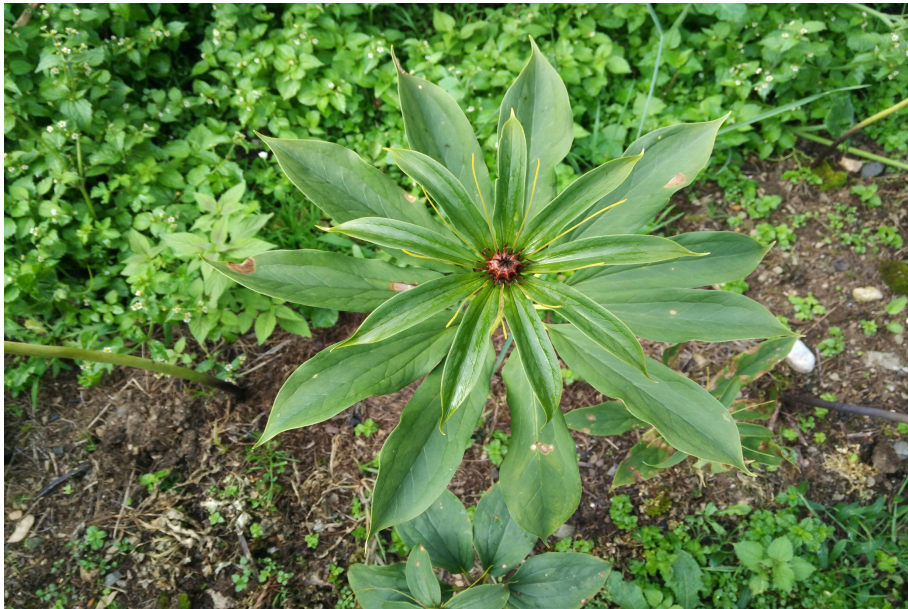

(B)

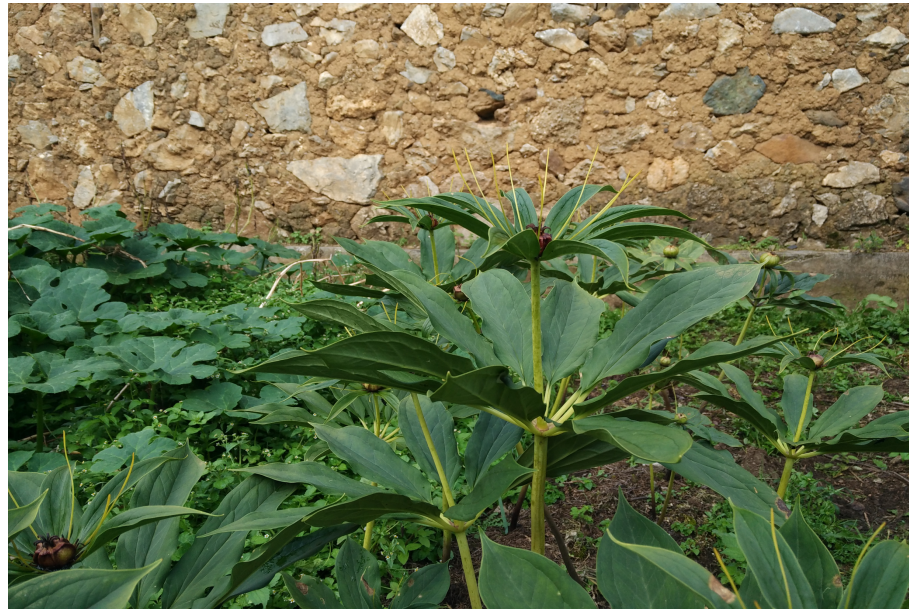

(C)

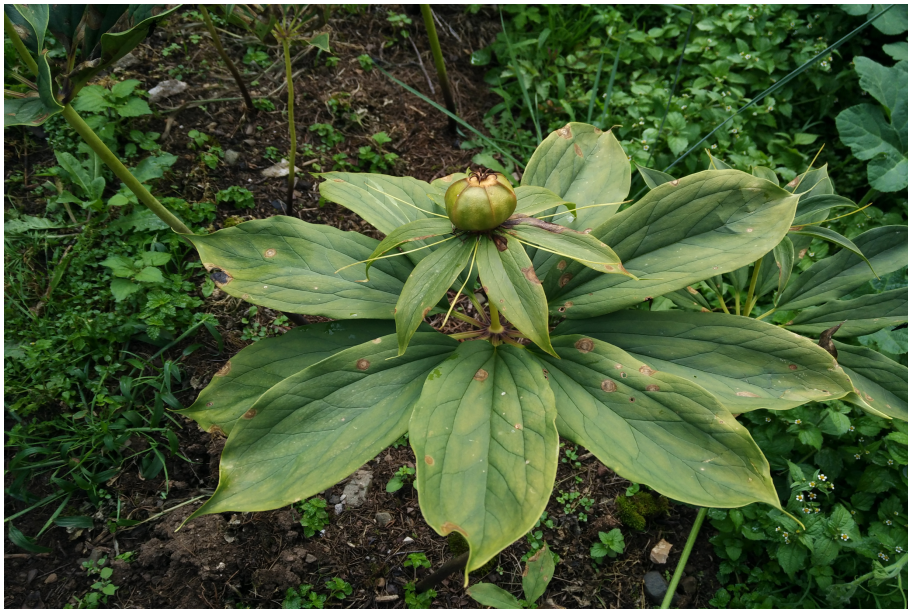

(D)

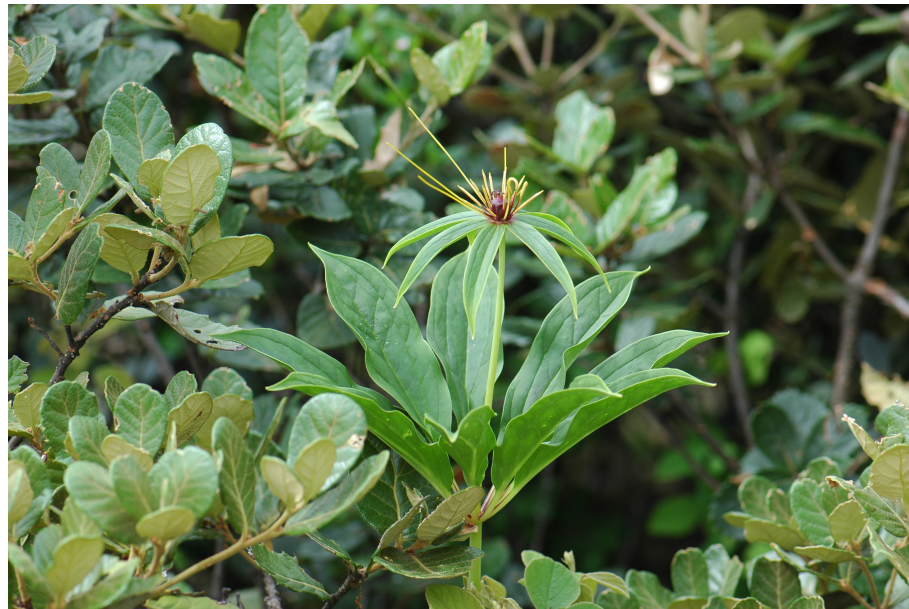

Supplement: Supplementary file 1 [file ECE3-9-10707-s001.pdf]

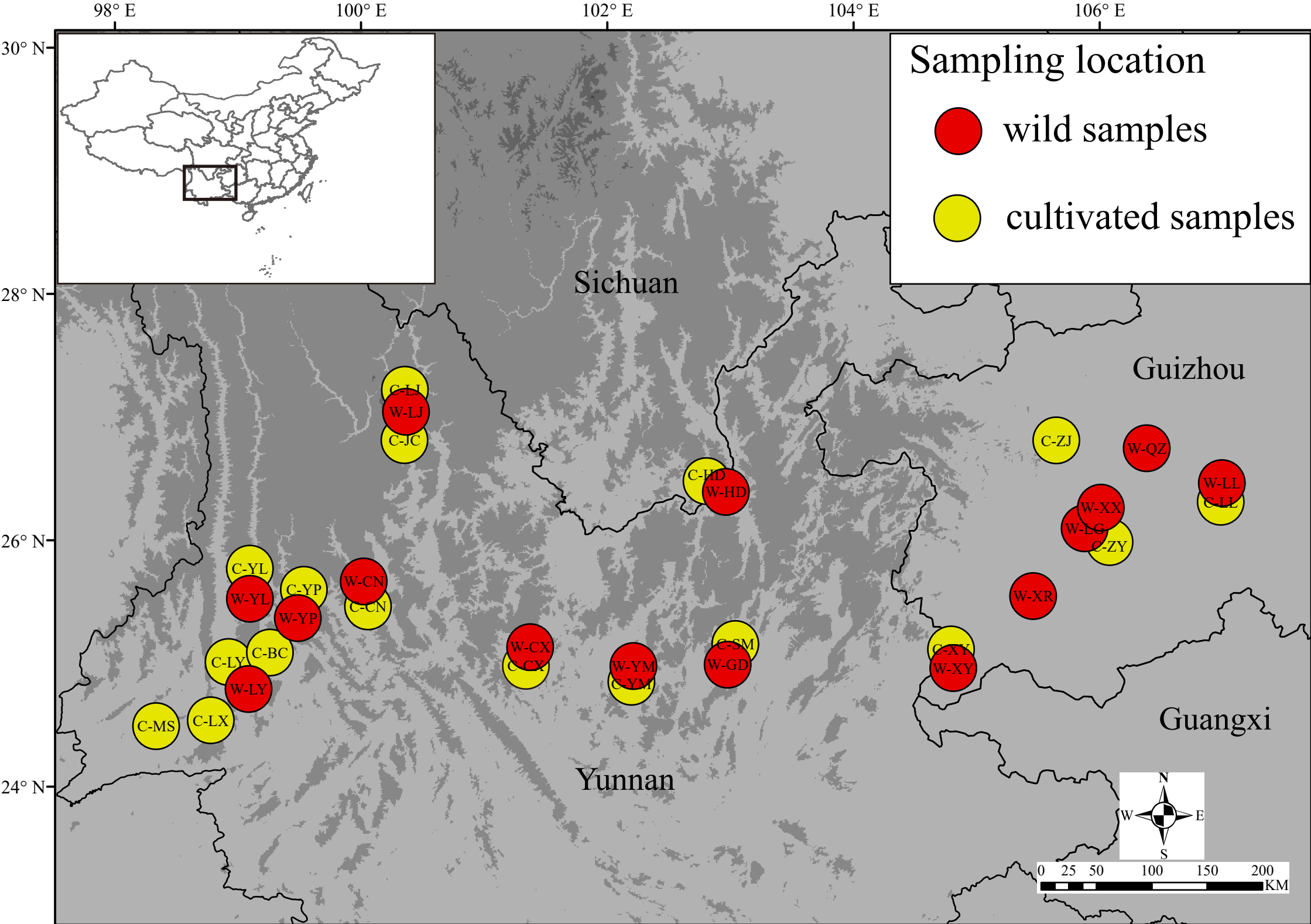

Supplement: Supplementary file 2 [file ECE3-9-10707-s002.pdf]

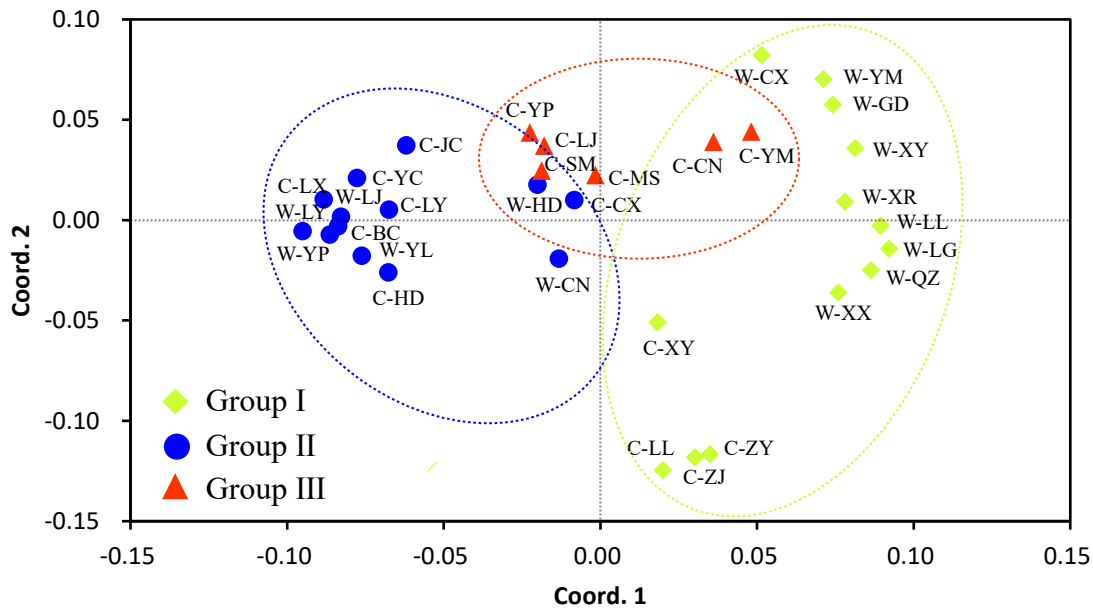

Supplement: Supplementary file 3 [file ECE3-9-10707-s003.pdf]

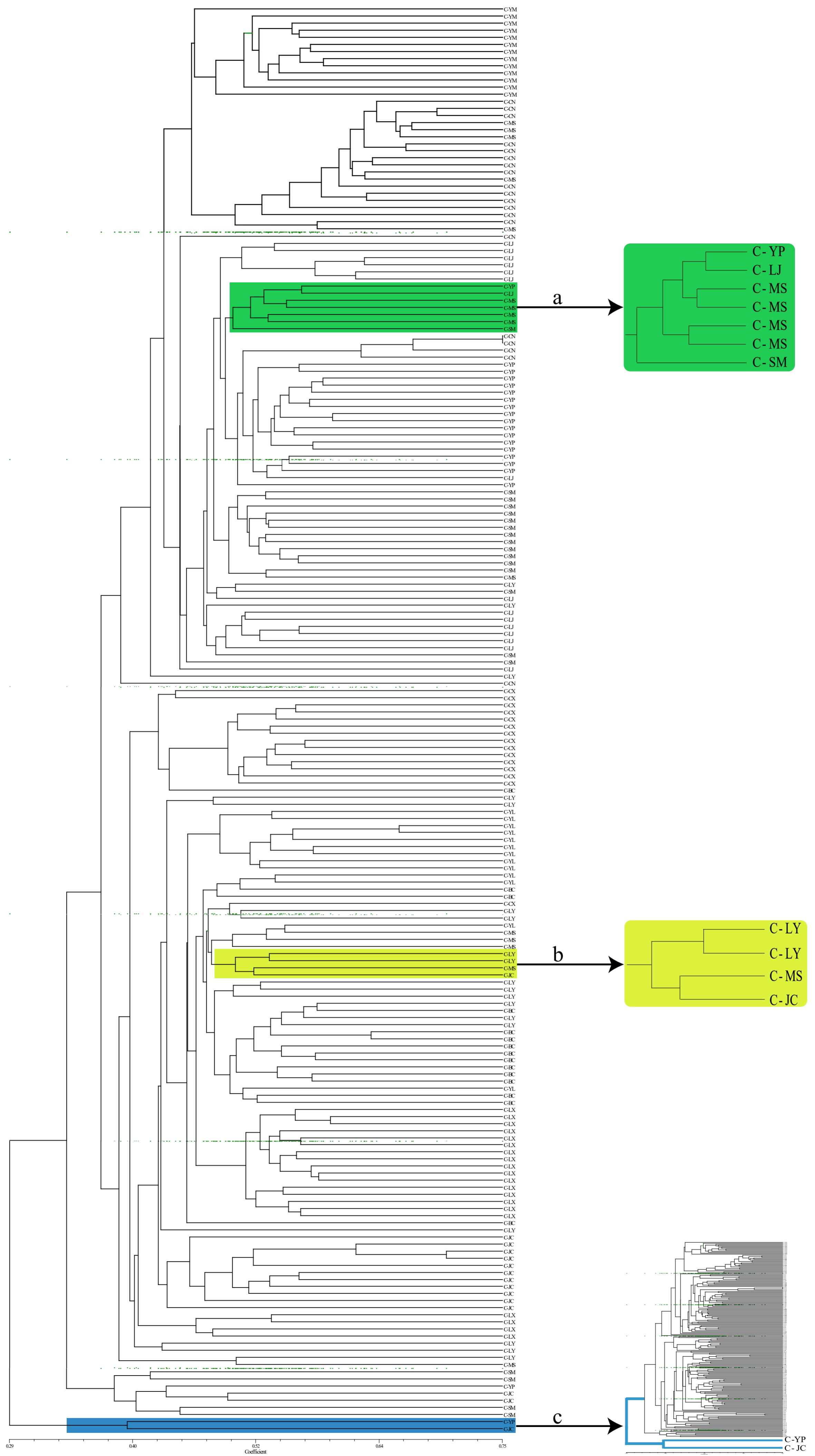

Supplement: Supplementary file 4 [file ECE3-9-10707-s004.pdf]
